# Long-term outcomes of bovine pericardium neo-aortic reconstructions in infected vascular fields

**DOI:** 10.1308/rcsann.2025.0116

**Published:** 2026-01-12

**Authors:** M Bakheet, T Babiker, I Zeynali, B Rawshdeh, G Riding, M Banihani

**Affiliations:** Lancashire Teaching Hospitals NHS Foundation Trust, UK

**Keywords:** Bovine pericardium, Aortic graft infection, Biological graft, Neo-aorta, Infected aortic field

## Abstract

**Introduction:**

Infected aortic fields are among the most complex surgical challenges, often requiring urgent intervention and associated with a high risk of morbidity and mortality. Bovine pericardium offers a customisable, off-the-shelf option for in situ reconstruction using biological material. We evaluate long-term outcomes following emergency bovine pericardium neo-aorta reconstruction in infected aortic fields.

**Methods:**

Prospectively collected data for all patients treated with bovine pericardium neo-aorta reconstructions from 2018 to date were analysed. The surgical approach included resection of the infected aortic segment, explantation of previous grafts and/or stents, and reconstruction of the neo-aorta using a bovine pericardium sheet. Data collected included patient demographics, comorbidities, clinical presentation, previous interventions, blood tests, complications, length of antibiotic treatment and long-term survival. All neo-aortas were enrolled in an annual ultrasound surveillance programme to monitor for aneurysmal degeneration.

**Results:**

Fifteen aortic reconstructions were performed. The most common indication was aorto-enteric fistulas (*n *= 7), followed by infected aortic grafts (*n *= 4), mycotic aneurysms (*n *= 3) and one contaminated field due to emergency colectomy. Median follow-up was 34 months (range 9–84). Thirty-day mortality was 7%, with another 7% at 10 weeks. Antibiotic-free survival rate was 86%. One patient (7%) required long-term antibiotics post-partial endovascular aneurysm repair explant. Complications included one graft occlusion with limb loss and one case of end-stage renal failure. No aneurysmal degeneration was identified during follow-up.

**Conclusion:**

Bovine pericardial neo-aorta reconstructions have shown excellent long-term resistance to infections and very good durability. Our data add to growing evidence supporting off-the-shelf use of bovine pericardium in emergency aortic reconstruction. Larger numbers through multicentre studies or special registries would help support more regular use.

## Introduction

Aortic infection, whether due to native aneurysm rupture, prosthetic graft infection or the development of an aorto-enteric fistula, represents a life-threatening pathology with significant surgical and perioperative risk and poor overall prognosis. Management typically necessitates complete excision of infected tissues followed by complex aortic reconstruction, often as an emergency.

The gold standard reconstruction using femoral veins is a lengthy operation that is best suited to fit patients and usually leads to significant morbidity. Cryopreserved grafts are not widely available for use, especially in emergencies, and are associated with a higher risk of degeneration and aneurysmal formation.^1^

Traditional synthetic materials used for these reconstructions, including commonly used rifampicin-soaked Dacron grafts, have shown multiple limitations, particularly an increased risk of reinfection or re-fistulisation, especially in contaminated fields, as highlighted in multiple series comparing them with biological alternatives.^2,3^

Recent guidelines from the European Society for Vascular Surgery highlight the complexity of managing aortic infections and emphasise that biological grafts offer significant advantages over synthetic alternatives, particularly in contaminated surgical fields, by reducing infection recurrence and improving long-term survival.^4^ Supporting this, a recent retrospective study by Melloni *et al* found that aortic repair using both preformed and home-made stapled xenopericardial grafts is safe and ensures durable, infection-free survival in the mid-term follow-up, underscoring the growing preference for biological grafts in contemporary practice.^5^

Bovine pericardial grafts can be easily fashioned into custom configurations intraoperatively and have demonstrated strong resistance to infection, owing to their biological composition and favourable integration with host tissue.^6,7^ Initial experiences and small series have reported reinfection rates between 5% and 16%. Almási-Sperling *et al* demonstrated similar outcomes in both peripheral and aortic fields.^8^ Recently, Glasgow *et al* compared bovine pericardial xenografts with autologous venous reconstruction, finding no significant difference in medium-term survival.^9^

Recent data have further reinforced these findings, with xenopericardial tube grafts and patches being successfully used in both native and prosthetic thoracic aorta infections, including cases with endocarditis and root involvement. Kondov *et al* and Kreibich *et al* highlighted the successful use of xenopericardial material in redo aortic root repairs and complex aortic infections, with acceptable morbidity and favourable infection control rates.^10,11^ Similarly, Anibueze *et al* demonstrated promising outcomes using xenoprosthetic neo-aortic grafts, with low reinfection rates and satisfactory 1-year survival in mycotic and graft-related aortic infections.^12^ A recent systematic review of xenoprosthetic grafts in thoracic and abdominal aortic infections by Grills *et al* showed a general good short-term outcome but relatively high median 30-day and late mortality of around 19%. They also highlighted the wide variation in follow-up with a focus on short-term follow-up in the majority of those studies. Notably, imaging follow-up was limited to 12 months.^13^ Most recently, Battistella *et al* compared xenopericardial grafts with cryopreserved allografts in native and graft-related abdominal aortic infections, showing comparable patency and infection-free survival, supporting wider application of xenograft-based reconstructions.^14^

This study aims to build on the available literature by presenting our institutional experience over 7 years using bovine pericardium in emergency reconstructions for infected aortic fields with full clinical and ultrasound scan-based long-term follow-up. Our objective is to evaluate both the clinical and structural outcomes associated with this approach, with a focus on infection resistance, graft durability, and complication rates.

## Methods

### Patient selection and clinical settings

All patients managed at our regional vascular centre between January 2018 and March 2024 who underwent emergency aortic reconstruction using bovine pericardial xenografts were prospectively enrolled into our unit’s database. Many were transferred from other regional hospitals because of the complexity of their presentations. Aortic field infection was confirmed by intraoperative findings of purulence, graft erosion or aorto-enteric fistula. Tissue culture results were variable.

Patient demographics, overall fitness, cardiac dysfunction, diabetes, renal impairment, malignancy, previous vascular interventions, details of surgical procedure, complications, intensive care stay, number of procedures, length of stay, 30-day mortality and regular follow-up outcomes were collected into a database with regular updates including blood results, clinical assessment and/or surveillance scans.

### Surgical technique

**Figure 1:**
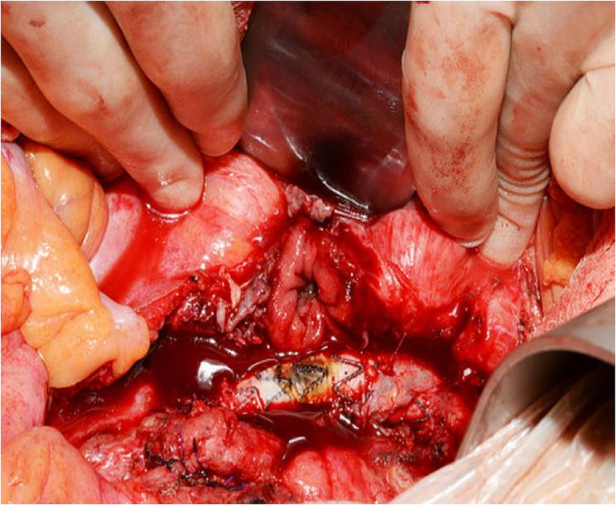
Explantation of previously implanted grafts during surgical exposure of the diseased aortic segment

**Figure 2:**
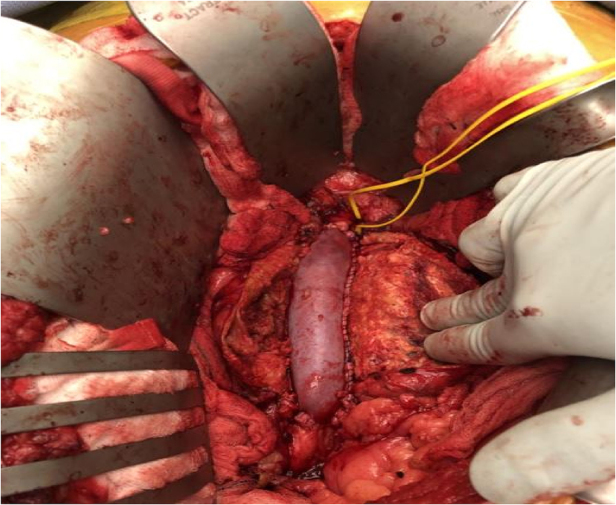
In situ reconstruction of the neo-aorta

The operative approach consisted of debridement of infected or devitalised tissue, including colon cancer resection in one case, explantation of previously implanted grafts or stents (Figure 1), and in situ reconstruction of the neo-aorta (Figure 2). Bovine pericardium sheets were tailored to shape using a continuous polypropylene suture and configured as needed either as straight tubes or bifurcated grafts. In the initial cases, an Omniflow biosynthetic graft was used for longer iliac limbs, but this was abandoned after two cases of early occlusion and replaced with full-length bovine pericardium reconstructed iliac limbs thereafter.

All patients received broad-spectrum antibiotics postoperatively, later tailored based on culture sensitivities. Duration of antibiotic cover was individualised for specific patient factors.

### Postoperative care and surveillance

Postoperatively, patients were managed early in the intensive care unit. Targeted antibiotic therapy was continued for a minimum of 6 weeks, with the duration individualised according to patient-specific factors. In selected cases, particularly for patients with partial explantation of endovascular aneurysm repair (EVAR) components, long-term suppressive antibiotics were prescribed. Microbiology input was routinely maintained throughout the treatment course until antibiotic therapy was discontinued.

All patients were enrolled in an annual surveillance programme that included clinical review and duplex ultrasonography to assess graft size, flow stability and detect subclinical complications. Inflammatory markers were monitored as part of the surveillance protocol for 4–6 weeks after cessation of antibiotic therapy to ensure sustained infection control.

Additional imaging, including computed tomography (CT) angiography, was performed when abnormalities were suspected or when the patient was already under CT surveillance for other diagnoses. Positron emission tomography (PET)–CT scanning was used selectively in cases in which imaging and clinical findings raised persistent suspicion of ongoing infection or indeterminate changes requiring further characterisation.

## Results

### Clinical presentation and operative indications

In total, 15 patients underwent emergency neo-aorta reconstruction. All presented with clinical or radiological evidence of infected aortic fields. The most common indication was for aorto-enteric fistulas (seven patients) and three of them had an added life-threatening gastrointestinal haemorrhage requiring massive transfusion protocols. Four patients had a confirmed infected prosthetic aortic graft infection, and three patients had mycotic aneurysms. Of those, two patients had rapid enlargement and sepsis and one had negative cultures despite significant inflammation and graft erosion. Only one case was in a clean infected aortic field during a simultaneous emergency colectomy for obstructing colonic malignancy.

### Follow-up and survival

The median follow-up period was 34 months (range 9–84). We had one case of 30-day mortality (7%), caused by multiorgan failure in the immediate postoperative period. Overall survival at 3 months was 93% (14 of 15 patients). One patient (7%) died at 10 weeks, from unrelated complications following discharge. Twelve patients stopped antibiotics 6 weeks postoperatively on average and only one patient required chronic suppressive antibiotics following partial removal of an infected EVAR stent that was re-stented as an emergency for massive gastrointestinal bleed.

### Long-term complications

One graft occlusion occurred at 12 months, resulting in critical limb ischaemia leading to above-knee amputation. One patient had renal failure that progressed to dialysis. This patient had a poor estimated glomerular filtration rate preoperatively with multiple comorbidities but was running out of antibiotic options. We observed no cases of aneurysmal degeneration on surveillance scans so far.

### Graft patency and durability

All bovine pericardium grafts remained patent throughout the follow-up period, with no evidence of thrombosis or structural failure. Surveillance imaging showed no dilation or degeneration of the pericardial conduits, affirming their long-term stability.

## Discussion

Our experience with bovine pericardial neo-aorta reconstruction supports its viability as an emergency solution in infected vascular fields. The favourable infection resistance, ease of use and intraoperative flexibility make it an attractive alternative to synthetic grafts, especially when time and availability are limiting factors.

The observed 30-day mortality of 7% is notably low given the severity and complexity of cases. Most patients recovered well postoperatively, with antibiotic-free survival exceeding 85%, aligning with previously published data and consistent with recent reports demonstrating the safety and durability of xenopericardial grafts in both thoracic and abdominal reconstructions.

Graft occlusion leading to limb loss occurred in a single patient. Interestingly, the worst outcome was for patients with aorto-bifemoral grafts with underlying occlusive peripheral vascular disease. By contrast, those with aneurysmal disease had better outcomes, highlighting the importance of atherosclerotic load in patient prognosis and systemic resilience.

Compared with synthetic grafts such as rifampicin-soaked Dacron, which carry higher reinfection risks, bovine pericardium offers a strong combination of safety, performance and practicality.^2,3^ Our results are in line with previously published systematic reviews and retrospective series, and further supported by a recent large European multicentre study by Weiss *et al*, which included 168 patients undergoing in situ reconstruction with custom-made bovine pericardial tube grafts across 18 centres.^4–6^ That study reported 1-year survival of 84%, reinfection-free survival of 96% and primary patency of 89%, closely matching our findings of 93% 3-month survival, 86% antibiotic-free survival and excellent long-term graft patency with no aneurysmal degeneration.^15^

The range of organisms identified across our patients reflects the complexity and unpredictability of infected aortic fields. We encountered common pathogens such as *Staphylococcus aureus* and *Escherichia coli*, but also more challenging organisms including *Candida albicans* and even *Mycobacterium tuberculosis* in one case. These findings illustrate the wide microbiological spectrum we face in clinical practice, often requiring early broad-spectrum antibiotics followed by tailored therapy. Managing fungal or tuberculosis-related infections brought additional layers of complexity, particularly because of the need for prolonged treatment and concerns around immunocompromise. Despite this, outcomes remained encouraging, further supporting the role of bovine pericardium as a resilient and reliable option even in the most hostile infectious environments.

Fortunately, primary aortic infection and aortic graft infections are rare, but that limits the numbers for a large-scale study of this intervention. Nevertheless, there is a growing literature that suggests a wider acceptance of this option in multiple centres with good short- and medium-term outcomes. Our series demonstrated prospectively the durability of those xenograft neo-aortas on long-term follow-up scans. However, life-long surveillance is needed to confirm such durability for all patients. If that is confirmed, then this type of repair might become an extra option in other clinical scenarios in the future.

### Study limitations

Like most studies in this field, the number of cases is typically small, which makes it difficult to draw strong generalisable conclusions. The sample reflects a highly selected group of complex, high-risk cases, and outcomes may differ in other settings or populations.

Follow-up was based on clinical assessments and annual ultrasound imaging, with a focus on patency, pseudo-aneurysm formation and aneurysmal degeneration.

Biosynthetic Omniflow graft was used for longer ilio-femoral extensions of the bovine pericardium tube graft in two cases. In both cases, the graft occluded early, and the type of graft in those cases could have affected the outcomes.

There was also some variation in the postoperative antibiotic regimen because it was individualised to patient factors, tissue culture results and the nature of infective organisms.

## Conclusions

Bovine pericardial xenografts are a safe, durable and infection-resistant option for aortic reconstruction in hostile, infected surgical fields. Their intraoperative adaptability and off-the-shelf availability are key advantages in emergency settings. Our single-centre data affirm their value in reducing mortality and reinfection, with preserved graft patency over extended follow-up.

We advocate for multicentre studies or national registries to validate these results on a larger scale and develop formalised guidelines for biological graft use in vascular infection management.

## Competing interests

The author/s declare no competing interests.

## Funding

The author/s received no financial support for the research, authorship and/or publication of this article.

## Ethical statement

All procedures were performed in accordance with the ethical standards of the institutional and national research committee.

## Consent to participate statement

Not applicable.

## Author contributions

**MEA Bakheet**: Formal analysis, Methodology, Project administration, Writing — original draft. **T Babiker**: Data curation. **I Zeynali**: Investigation, Resources. **B Rawshdeh**: Data curation, Validation. **G Riding**: Conceptualisation, Resources. **M Banihani**: Supervision, Writing — review & editing.

## Artificial Intelligence

The author/s declare that no AI was used to conduct the study or prepare the manuscript.
